# Co‐infection analysis of bacterial and viral respiratory pathogens from clinically healthy swine in Eastern China

**DOI:** 10.1002/vms3.533

**Published:** 2021-05-25

**Authors:** Haodan Zhu, Xinjian Chang, Jinzhu Zhou, Dandan Wang, Junming Zhou, Baochao Fan, Yanxiu Ni, Jie Yin, Lixin Lv, Yongxiang Zhao, Kongwang He, Bin Li

**Affiliations:** ^1^ Institute of Veterinary Medicine Key Laboratory of Veterinary Biological Engineering and Technology Ministry of Agriculture Jiangsu Academy of Agricultural Sciences Nanjing China; ^2^ Jiangsu Co‐Innovation Center for the Prevention and Control of Important Animal Infectious Disease and Zoonose Yangzhou University Yangzhou PR China; ^3^ Jiangsu Key Laboratory for Food Quality and Safety‐State Key Laboratory Cultivation Base of Ministry of Science and Technology Nanjing China; ^4^ College of Animal Science and Technology Inner Mongolia University for Nationalities Tongliao China; ^5^ School of Food and Biological Engineering Jiangsu University Zhenjiang China

**Keywords:** co‐infection, molecular detection, pigs, porcine respiratory disease complex, respiratory pathogens

## Abstract

Porcine respiratory disease complex (PRDC) is one of the most challenging health concerns for pig production worldwide. The aim of the present study was to determine the prevalence of pathogens associated with PRDC, including porcine reproductive and respiratory syndrome virus (PRRSV) and porcine circovirus type 2 (PCV2) and bacterial agents, such as *Streptococcus* *suis*, *Haemophilus parasuis* and *Actinobacillus pleuropneumoniae*, in clinically healthy pigs in Eastern China. Molecular detection revealed positive single‐pathogen detection rates of 59.9%, 27.2%, 52.3%, 33.2% and 0.4% for PCV2, PRRSV, *S*. *suis*, *H*. *parasuis* and *A*. *pleuropneumoniae*, respectively. Co‐infection with more than one pathogen was frequently detected in these samples, with PCV2/*S*. *suis*, *H*. *parasuis* and PCV2/*H*. *parasuis* mixed infection rates of 35.4%, 33.2% and 21.6%, respectively, and PCV2/*S*. *suis*/*H*. *parasuis* and PRRSV/PCV2/*S*. *suis* co‐infection rates of 21.6% and 6.2%, respectively. These results suggest that mixed infections are prevalent among PRDC cases in swine, which may pose a greater threat to the health of herds compared with single‐pathogen infections.

## Graphical abstract

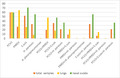

A broad range of pathogens is capable of causing respiratory diseases in pigs, with most cases found to be polymicrobial and multifactorial (Chae, 2016; Opriessnig et al., 2011). Because the predominant pathogens can vary significantly among production sites, the combined causative factors are referred to as porcine respiratory disease complex (PRDC). Generally, pathogens within the PRDC trigger lung damage, which in turn results in low economic efficiency, poor growth rates and higher medication and management costs (Fablet, Marois‐Créhan, et al., 2012). Bacterial pathogens most frequently detected in PRDC cases include *Actinobacillus pleuropneumoniae*, *Streptococcus suis* and *Haemophilus parasuis* (Cheong et al., 2017; Fablet, Marois, et al., 2012), while predominant viral pathogens include porcine reproductive and respiratory syndrome virus (PRRSV) and porcine circovirus type 2 (PCV2) (Fablet, Marois‐Créhan, et al., 2012). Based on their ability to induce lesions in respiratory tissues, pathogens associated with PRDC can be categorised as primary, secondary or opportunistic infectious agents (Dorr et al., 2007).

To date, several interactions and synergisms between respiratory pathogens have been described (Li et al., 2017). For example, several infectious agents with low pathogenicity can together result in high‐grade respiratory diseases (Opriessnig et al., 2011). Based on these observations, the present study aimed to survey viral (PCV2 and PRRSV) and bacterial (*S*. *suis*, *H*. *parasuis* and *A*. *pleuropneumoniae*) co‐infections in nasal swab samples and the lungs of healthy pigs which were collected from Eastern China from 2017 to 2019.

For this study, a total of 898 samples were collected from clinically healthy pigs from 27 farms in Eastern China. Nasal swab samples (*n* = 522) were collected from piglets from 15 farms, while lung samples (*n* = 376) were randomly collected from finishing pigs from 12 farms at the slaughterhouse.

To detect bacterial pathogens, the surface of each lung was seared and a tissue sample aseptically collected. Samples were inoculated into 1.5 ml of Todd–Hewitt broth medium containing 0.02% nicotinamide adenosine dinucleotide (NAD) and incubated with gentle shaking for 2–4 hr, as described previously (MacInnes et al., 2008). Nasal swabs were cultured in the same way. Following incubation, cells were pelleted by centrifugation and resuspended in 100 μL of sterile ddH_2_O. Suspensions were then boiled for 10 min before being incubated on ice for a further 10 min. Following centrifugation, supernatants were transferred to fresh tubes and stored at −80°C for use as templates for polymerase chain reaction (PCR)‐based detection assays.

For virus detection, 0.1 g lung tissue samples were homogenised using an MP FastPrep‐24 Homogeniser (MP Biomedicals, Irvine, CA, USA), whereas nasal swab samples were placed in 2‐mL sterile plastic tubes containing 1.5 ml of sterile phosphate‐buffered saline (PBS, pH = 7.2). DNA and RNA were then extracted from all samples using DNAout and RNAout kits (Tiandz, Beijing, China), respectively, as per the manufacturer's instructions. Extracted DNA and RNA were stored at −80°C.

For *S*. *suis* detection, a PCR targeting a fragment of 689 bp of *gdh* was used (Gottschalk et al., 2013). Detection of *H*. *parasuis* was based on the amplification of an 821 bp fragment of the 16SrRNA gene. The APP DNA was identified by using a pair of specific primers for Apx‐IV gene (MacInnes et al., 2008). For PCV2 detection, a PCR was carried out using primers previously described (Jiang et al., 2017). RT‐PCR‐targeting ORF5 was used for the detection of PRRSV (Peng et al., 2017). Primers used in this study are listed in Table 1.

**TABLE 1:** Primers used for PCR‐based detection of pathogens associated with PRDC

Primer	Target gene	Primer Sequences（5’–3’^，^）	Products Size	Reference
PCV2‐F	ORF2	TTCGGTACCAGCTATGACGTATCCAAG	751bp	(Jiang et al., 2017)
PCV2‐R		GCCAAGCTTTCACTTCGTAATGGTTTT		
PRRSV‐F	ORF5	TGGCAATTTGAATGTTCAAGTATG	681bp	(Peng et al., 2017)
PRRSV‐R		CTGTGCTATCATTGCAGAAGTCGT		
SS‐F	GDH	GCAGCGTATTCTGTCTTTCG	689bp	(Gottschalk et al., 2013)
SS‐R		CCATGGACAGATAAACTAGG		
HPS‐F	16SrRNA	GTGATGAGGAAGGGTGGTGT	822bp	(MacInnes et al., 2008)
HPS‐R		GGCTTCGTCACCCTCTGT		
APP‐F	Apx‐IV	TTATCCGAACTTTGGTTTAGCC	417bp	(MacInnes et al., 2008)
APP‐R		CATATTTGATAAAACCATCCGTC		

Pathogen detection results for the 898 samples are presented in Table 2 and Figure 1. Samples were screened for typical swine respiratory viruses PRRSV and PCV2 and common swine respiratory bacteria *S*. *suis*, *H*. *parasuis* and *A*. *pleuropneumoniae*. Overall, single‐pathogen detection rates for PCV2, PRRSV, *S*. *suis*, *H*. *parasuis* and *A*. *pleuropneumoniae* were 59.9%, 27.2%, 52.3%, 33.2% and 0.4%, respectively. PCV2/*S*. *suis* was the most frequently detected combination of infectious agents among all samples (35.4%), followed by *H*. *parasuis*/*S*. *suis* co‐infection (33.2%) and PCV2/*H*. *parasuis* coinfection (21.6%). The most prevalent multi‐pathogen infections were PCV2/*S*. *suis*/*H*. *parasuis* (21.6%) and PRRSV/PCV2/*S*. *suis* (6.2%).

**TABLE 2:** Detection rates for PRDC pathogens in nasal swab samples and lung samples

Pathogen	Total prevalence (*n* = 898)		Lungs samples (*n* = 376)		Nasal swabs (*n* = 522)	
	*N*	%	*N*	%	*N*	%
PCV2	538	59.9	228	60.6	310	59.4
PRRSV	244	27.2	244	64.9	0	0
*S.suis*	470	52.3	100	26.6	370	71
*H. parasuis*	298	33.2	8	8.5	290	55.6
*A. pleuropneumoniae*	4	0.4	4	1.1	0	0
PCV2 + PRRSV	180	20	180	47.8	0	0
PCV2 + *S.suis*	318	35.4	68	18.1	250	47.9
PCV2 + *H*. *parasuis*	194	21.6	4	1.1	190	36.4
PCV2 *+ A*. *pleuropneumoniae*	0	0	0	0	0	0
PRRSV + S*.suis*	80	8.9	80	21.3	0	0
PRRSV + H. *parasuis*	4	0.4	4	1.1	0	0
PRRSV + A. *pleuropneumoniae*	4	0.4	4	1.1	0	0
*S.suis* + H. *parasuis*	298	33.2	8	2.1	290	55.6
*S.suis* + A. *pleuropneumoniae*	0	0	0	0	0	0
*H. parasuis* + A. *pleuropneumoniae*	0	0	0	0	0	0
PCV2 + PRRSV + S*.suis*	56	6.2	56	14.9	0	0
PCV2 + PRRSV + H. *parasuis*	4	0.4	4	1.1	0	0
PCV2 + PRRSV + A. *pleuropneumoniae*	0	0	0	0	0	0
PCV2 + *S.suis* + H. *parasuis*	194	21.6	4	1.1	190	36.4
PCV2 + *S.suis* + A. *pleuropneumoniae*	0	0	0	0	0	0
PCV2 + *H*. *parasuis* + A. *pleuropneumoniae*	0	0	0	0	0	0

**FIGURE 1:**
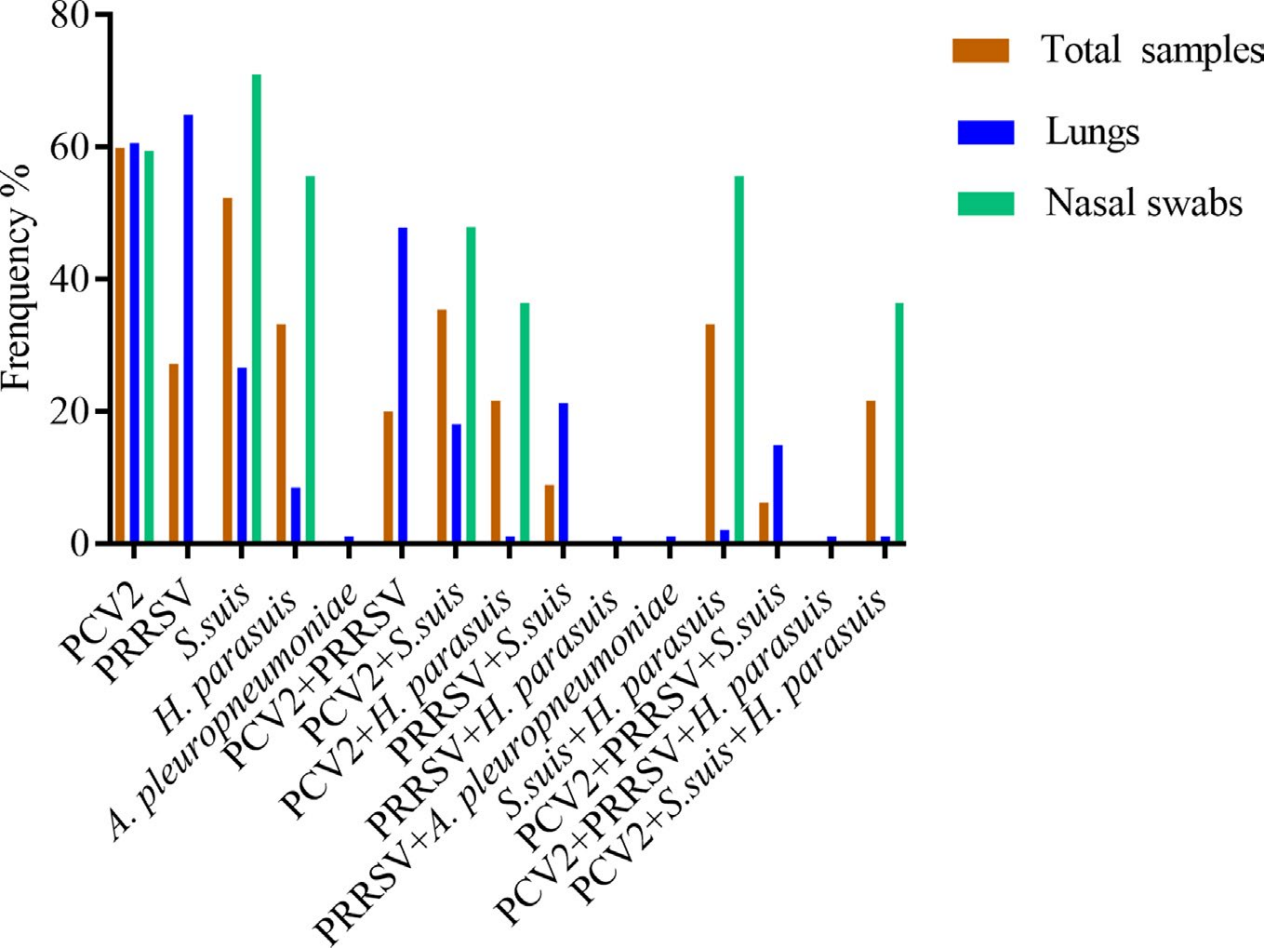
Prevalence of major pathogens associated with PRDC

Pathogen detection rates for each sample type are presented in Table 2 and Figure 1. Overall, more than 60% of lung tissue samples were PCR positive for viruses, while bacterial detection rates ranging from 1.1% to 26.6%. PRRSV/PCV2 co‐infection was most prevalent among the lung tissue samples (47.8%), followed by PRRSV/*S*. *suis* co‐infection (21.3%) and PCV2/*S*. *suis* co‐infection (18.1%). Multi‐pathogen infection (PRRSV/PCV2/*S*. *suis*) was detected in 56 (14.9%) lung tissue samples.

Among the nasal swab samples, *S*. *suis*, PCV2 and *H*. *parasuis* were detected in 71%, 59.4% and 55.6% of samples, respectively. Neither PRRSV nor *A*. *pleuropneumoniae* was detected in nasal swab samples in this study. *S*. *suis*/*H*. *parasuis* coinfection was the predominant mixed infection among the nasal samples (55.6%), followed by PCV2/*S*. *suis* co‐infection (47.9%) and PCV2/*H*. *parasuis* co‐infection (36.4%). PCV2/*S*. *suis*/*H*. *parasuis* multi‐pathogen infection was observed in 190 nasal swab samples (36.4%). Overall, bacterial coinfection was more frequently observed than viral co‐infection in the nasal swab samples.

The most prevalent pathogen combinations among all 898 samples based on PCR data are summarised in Figure 2. Our results suggested that co‐infection/multi‐pathogen infection was more common than single‐pathogen infection among pigs in Eastern China.

**FIGURE 2:**
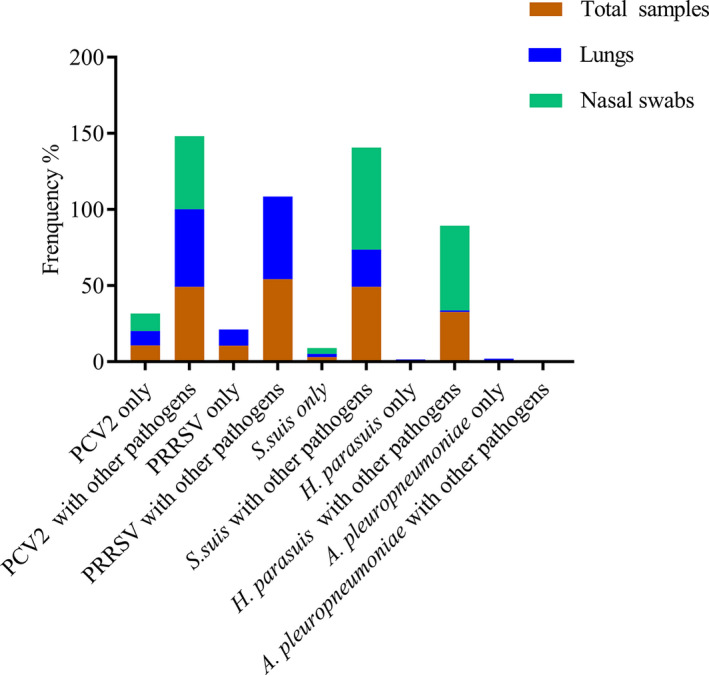
Number of samples containing major pathogens and major pathogen combinations

PRDC is one of the most important health issues impacting pig production. Knowledge of the pathogens associated with PRDC is critical for the implementation of prevention and control measures. Overall, 67% (602/898) and 52.8% (474/898) of samples in the current study were PCR positive for respiratory viruses and bacteria, respectively, with PCV2 (59.9%) and *S*. *suis* (53.2%) the most frequently detected viral and bacterial pathogens, respectively. Previous studies have revealed varying rates of PRDC pathogens depending on the sample type and sampling method. A study by O’Sullivan et al. (O'Sullivan et al., 2011) revealed prevalence rates for *S*. *suis*, PRRSV and PCV2 in tonsil samples from pigs at slaughter in Canada of 53.7%, 22.0% and 11.9%, respectively. Similarly, Fablet et al. (Fablet et al., 2011) recorded prevalence rates of 67.1%, 30.9% and 23.4% for *S*. *suis*, *A*. *pleuropneumoniae* and *H*. *parasuis* among nasal, tonsillar and oropharyngeal swab samples from pigs in France. However, other studies have revealed lower prevalence rates, with *S*. *suis*, *H*. *parasuis* and *A*. *pleuropneumoniae* observed in only 17.4%, 29.4% and 5.9% of bronchial swab samples and 14.7%, 50% and 2.9% of lung tissue samples, respectively, from pigs in Germany (Moorkamp et al., 2008). Interestingly, in a study comparing pathogens in healthy pigs with those in pigs with pneumonia showed that PRRSV, PCV2 and *H*. *parasuis* were prevalent among all samples regardless of the presence/absence of clinical symptoms (Palzer et al., 2008). The high detection rates observed in the current study suggest that respiratory pathogens are prevalent among herds in Eastern China.

Co‐infections in various combinations were observed in a high percentage of the lung samples and nasal swabs in the current study. Although this is the first report of PCV2/*S*. *suis* co‐infection, previous studies have reported co‐infections caused by combinations of PCV2, PRRSV and *A*. *pleuropneumoniae* (Dorr et al., 2007). In such cases, infected hosts were then vulnerable to opportunistic infection by other pathogens (Pogranichniy et al., 2002). A similar tendency was observed in the current study, with single‐pathogen detection rates ranging from 0% to 10.7% but co‐infection rates (with one or more pathogens) increasing to 32.8%–54.3% (Figure 2). Statistically significant correlations between pathogens are of particular concern for PRDC management. In the current study, significant correlations between PCV2 and *S*. *suis*, PRRSV and *S*. *suis*, PRRSV and *H*. *parasuis* and *S*. *suis* and *H*. *parasuis* were identified. However, it was not possible to analyse the association between *A*. *pleuropneumoniae* and other pathogens because of the small number of positive samples. The identification of these significant pathogen pairs indicates that if one pathogen is frequently detected within a herd, that herd may also be vulnerable to co‐infection with the corresponding paired pathogen.

In conclusion, this is the first investigation into the prevalence of PRDC pathogens in Eastern China. The results of this study provide a better understanding of the interactions between major bacterial and viral PRDC pathogens, which will aid in disease prevention and treatment.

## CONFLICT OF INTEREST

No potential conﬂict of interest was reported by the authors.

## AUTHOR CONTRIBUTION

**Haodan Zhu:** Data curation; Investigation; Methodology; Writing‐original draft; Writing‐review & editing. **Xinjian Chang:** Data curation; Formal analysis; Methodology. **Jinzhu Zhou:** Data curation; Methodology; Resources. **Dandan Wang:** Data curation; Formal analysis; Investigation; Methodology; Resources. **Junming Zhou:** Data curation; Formal analysis; Investigation; Methodology; Resources; Supervision. **Baochao Fan:** Conceptualization; Data curation; Project administration; Resources; Validation. **Jie Yin:** Data curation; Formal analysis; Resources. **Yongxiang Zhao:** Data curation; Formal analysis; Methodology; Project administration; Resources. **Kongwang He:** Data curation; Supervision; Visualization.

### PEER REVIEW

The peer review history for this article is available at https://publons.com/publon/10.1002/vms3.533.

## References

[vms3533-bib-0001] Chae, C. (2016). Porcine respiratory disease complex: Interaction of vaccination and porcine circovirus type 2, porcine reproductive and respiratory syndrome virus, and Mycoplasma hyopneumoniae. The Veterinary Journal, 22, 1–6. 10.1016/j.tvjl.2015.10.030 27256017

[vms3533-bib-0002] Cheong, Y., Oh, C., Lee, K., & Cho, K. H. (2017). Survey of porcine respiratory disease complex‐associated pathogens among commercial pig farms in Korea via oral fluid method. Journal of Veterinary Science, 18, 283–289. 10.4142/jvs.2017.18.3.283 27586468 PMC5639080

[vms3533-bib-0003] Dorr, P. M., Baker, R. B., Almond, G. W., Wayne, S. R., & Gebreyes, W. A. (2007). Epidemiologic assessment of porcine circovirus type 2 coinfection with other pathogens in swine. Journal of the American Veterinary Medical Association, 230(2), 244–250. 10.2460/javma.230.2.244 17223759

[vms3533-bib-0004] Fablet, C., Marois, C., Dorenlor, V., Eono, F., Eveno, E., Jolly, J. P., Le Devendec, L., Kobisch, M., Madec, F., & Rose, N. (2012). Bacterial pathogens associated with lung lesions in slaughter pigs from 125 herds. Research in Veterinary Science, 93(2), 627–630. 10.1016/j.rvsc.2011.11.002 22133708

[vms3533-bib-0005] Fablet, C., Marois, C., Kuntz‐Simon, G., Rose, N., Dorenlor, V., Eono, F., Eveno, E., Jolly, J. P., Le Devendec, L., Tocqueville, V., Quéguiner, S., Gorin, S., Kobisch, M., & Madec, F. (2011). Longitudinal study of respiratory infection patterns of breeding sows in five farrow‐to‐finish herds. Veterinary Microbiology, 147, 329–339. 10.1016/j.vetmic.2010.07.005 20696539 PMC7117213

[vms3533-bib-0006] Fablet, C., Marois‐Créhan, C., Simon, G., Grasland, B., Jestin, A., Kobisch, M., Madec, F., & Rose, N. (2012). Infectious agents associated with respiratory diseases in 125 farrow‐to‐finish pig herds: A cross‐sectional study. Veterinary Microbiology, 157(1‐2), 152–163. 10.1016/j.vetmic.2011.12.015 22226820

[vms3533-bib-0007] Gottschalk, M., Lacouture, S., Bonifait, L., Roy, D., Fittipaldi, N., & Grenier, D. (2013). Characterization of Streptococcus suis isolates recovered between 2008 and 2011 from diseased pigs in Quebec. Canada Veterinary Microbiology, 162, 819–825. 10.1016/j.vetmic.2012.10.028 23177911

[vms3533-bib-0008] Jiang, C. G., Wang, G., Tu, Y. B., Liu, Y. G., Wang, S. J., Cai, X. H., & An, T. Q. (2017). Genetic analysis of porcine circovirus type 2 in China. Archives of Virology, 162(9), 2715–2726. 10.1007/s00705-017-3414-1 28578523

[vms3533-bib-0009] Li, J., Wang, S., Li, C., Wang, C., Liu, Y., Wang, G., He, X., Hu, L., Liu, Y., Cui, M., Bi, C., Shao, Z., Wang, X., Xiong, T., Cai, X., Huang, L., & Weng, C. (2017). Secondary Haemophilus parasuis infection enhances highly pathogenic porcine reproductive and respiratory syndrome virus (HP‐PRRSV) infection‐mediated inflammatory responses. Veterinary Microbiology, 204, 35–42. 10.1016/j.vetmic.2017.03.035 28532803

[vms3533-bib-0010] MacInnes, J. I., Gottschalk, M., Lone, A. G., Metcalf, D. S., Ojha, S., Rosendal, T., Watson, S. B., & Friendship, R. M. (2008). Prevalence of Actinobacillus pleuropneumoniae, Actinobacillus suis, Haemophilus parasuis, Pasteurella multocida, and Streptococcus suis in representative Ontario swine herds. Canadian Journal of Veterinary Research, 72, 242–248. 18505187 PMC2327245

[vms3533-bib-0011] Moorkamp, L., Nathues, H., Spergser, J., Tegeler, R., & Grosse Beilage, E. (2008). Detection of respiratory pathogens in porcine lung tissue and lavage fluid. The Veterinary Journal, 175, 273–275. 10.1016/j.tvjl.2007.01.008 17339121

[vms3533-bib-0012] Opriessnig, T., Gimenez‐Lirola, L. G., & Halbur, P. G. (2011). Polymicrobial respiratory disease in pigs. Animal Health Research Reviews, 12, 133–148. 10.1017/S1466252311000120 22152290

[vms3533-bib-0013] O'Sullivan, T., Friendship, R., Blackwell, T., Pearl, D., McEwen, B., Carman, S., Slavić, D., & Dewey, C. (2011). Microbiological identification and analysis of swine tonsils collected from carcasses at slaughter. Canadian Journal of Veterinary Research, 75, 106–111. 21731180 PMC3062919

[vms3533-bib-0014] Palzer, A., Ritzmann, M., Wolf, G., & Heinritzi, K. (2008). Associations between pathogens in healthy pigs and pigs with pneumonia. Veterinary Record, 162, 267–271. 10.1136/vr.162.9.267 18310558

[vms3533-bib-0015] Peng, Z., Zhao, T., Liang, W., Song, W., Gao, Z., Tang, X., Chen, H., & Wu, B. (2017). RT‐PCR detection of porcine reproductive and respiratory syndrome virus based on the ORF5 gene in mainland China, 2012–2015. Acta Virologica, 61(03), 336–340. 10.4149/av_2017_312 28854798

[vms3533-bib-0016] Pogranichniy, R. M., Yoon, K. J., Harms, P. A., Sorden, S. D., & Daniels, M. (2002). Case–control study on the association of porcine circovirus Type 2 and other swine viral pathogens with postweaning multisystemic wasting syndrome. Journal of Veterinary Diagnostic Investigation, 14(6), 449–456. 10.1177/104063870201400601 12423025

